# Revisiting and corrections to the annotated SRSF3 (SRp20) gene structure and RefSeq sequences from the human and mouse genomes

**DOI:** 10.1016/j.cellin.2023.100089

**Published:** 2023-02-26

**Authors:** Lulu Yu, Vladimir Majerciak, Rong Jia, Zhi-Ming Zheng

**Affiliations:** a Tumor Virus RNA Biology Section, HIV Dynamics and Replication Program, Center for Cancer Research, National Cancer Institute, Frederick, MD, 21702, USA; b The State Key Laboratory Breeding Base of Basic Science of Stomatology (Hubei-MOST), Key Laboratory of Oral Biomedicine Ministry of Education, School & Hospital of Stomatology, Wuhan University, Wuhan, China

**Keywords:** SRSF3, Genome structure, Gene expression, RNA isoforms, 5ʹ UTR, 3ʹ UTR

## Abstract

SRSF3 (SRp20) is the smallest member of the serine/arginine (SR)-rich protein family. We found the annotated human SRSF3 and mouse Srsf3 RefSeq sequences are much larger than the detected SRSF3/Srsf3 RNA size by Northern blot. Mapping of RNA-seq reads from various human and mouse cell lines to the annotated SRSF3/Srsf3 gene illustrated only a partial coverage of its terminal exon 7. By 5ʹ RACE and 3ʹ RACE, we determined that SRSF3 gene spanning over 8422 bases and Srsf3 gene spanning over 9423 bases. SRSF3/Srsf3 gene has seven exons with exon 7 bearing two alternative polyadenylation signals (PAS). Through alternative PAS selection and exon 4 exclusion/inclusion by alternative RNA splicing, SRSF3/Srsf3 gene expresses four RNA isoforms. The major SRSF3 mRNA isoform with exon 4 exclusion by using a favorable distal PAS to encode a full-length protein is 1411 nt long (not annotated 4228 nt) and the same major mouse Srsf3 mRNA isoform is only 1295 nt (not annotated 2585 nt). The difference from the redefined RNA size of SRSF3/Srsf3 to the corresponding RefSeq sequence is at the 3’ UTR region. Collectively, the redefined SRSF3/Srsf3 gene structure and expression will allow better understanding of SRSF3 functions and its regulations in health and diseases.

## Highlights

• Human SRSF3 and mouse Srsf3 are oversized in the annotated NCBI RefSeq sequences.
• SRSF3 and Srsf3 RNA are primarily ∼1.5 kb in size, ∼2.8 kb and ∼1.3 kb shorter than the respective RefSeq sequence.
• The redefined 3′ UTR contributes to the observed difference from the RefSeq sequence.
• Four RNA isoforms of SRSF3/Srsf3 are expressed by alternative RNA splicing and polyadenylation.

## Graphical abstract

**Figure:**
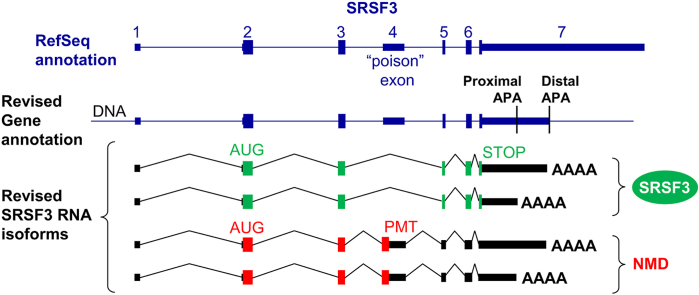

## 1 Introduction

The serine/arginine rich proteins (SR proteins) are members of key splicing factors involved in regulating pre-mRNA splicing (Zahler et al., 1992; Manley and Krainer, 2010), which contain one or two N-terminal RNA recognition motifs (RRM) and a C-terminal arginine- and serine-rich (RS) domain. A total of 12 classical SR splicing factors (SRSFs), namely SRSF1-SRSF12, have been reported. SRSF3, also called SRp20 or SFRS3, is the smallest member of the SR protein family. Human SRSF3 and mouse Srsf3 (X16) initially discovered in 1991 (Roth et al., 1991; Ayane et al., 1991) are highly conserved.

In addition to its important role in alternative RNA splicing (Jia et al., 2009, 2019; Majerciak et al., 2014; Ajiro et al., 2016), SRSF3 also plays an important role in RNA polyadenylation (Lou et al., 1998; Maciolek and McNally, 2007), RNA export (Huang and Steitz, 2001; Huang et al., 2003), pri-miRNA processing (Auyeung et al., 2013; Ajiro et al., 2016; Kim et al., 2018) and protein translation (Fitzgerald and Semler, 2011; Kim et al., 2014). Mouse embryos lacking SRSF3 do not form a blastocyst (Jumaa et al., 1999), and more importantly, we and others found that SRSF3 is tumorigenic acting as a proto-oncogene when overexpressed and is frequently upregulated in various types of cancer (Jia et al., 2010; He et al., 2004, 2011). The genome-wide analyses in a mouse tumor cell line (Ankö et al., 2010) and a human osteosarcoma cell line (Ajiro et al., 2016) have identified many RNA targets of SRSF3. Together, these observations indicate that SRSF3 is one of the master regulators of RNA processing and cell biology which aberrant expression leads to carcinogenesis. In the course of investigating how SRSF3 involves in oncogenesis, we discovered the gene structure of SRSF3 and its reference sequence (RefSeq) from the NCBI RefSeq NM_003017.5 for human SRSF3 and NM_013663.5 for mouse Srsf3 are incorrectly annotated with a size of 4228 nt and 2585 nt, respectively. In the present study, we experimentally reanalyzed SRSF3/Srsf3 gene structure and its expression in human and mouse cells. Based on our findings, we report the re-defined gene structure of SRSF3/Srsf3 and the corrected SRSF3/Srsf3 RNA isoforms and their sequences.

## 2 Results

### 2.1 Authentic human SRSF3 and mouse Srsf3 RNA transcripts are much shorter than the NCBI annotated RefSeq size

**Fig. 1:**
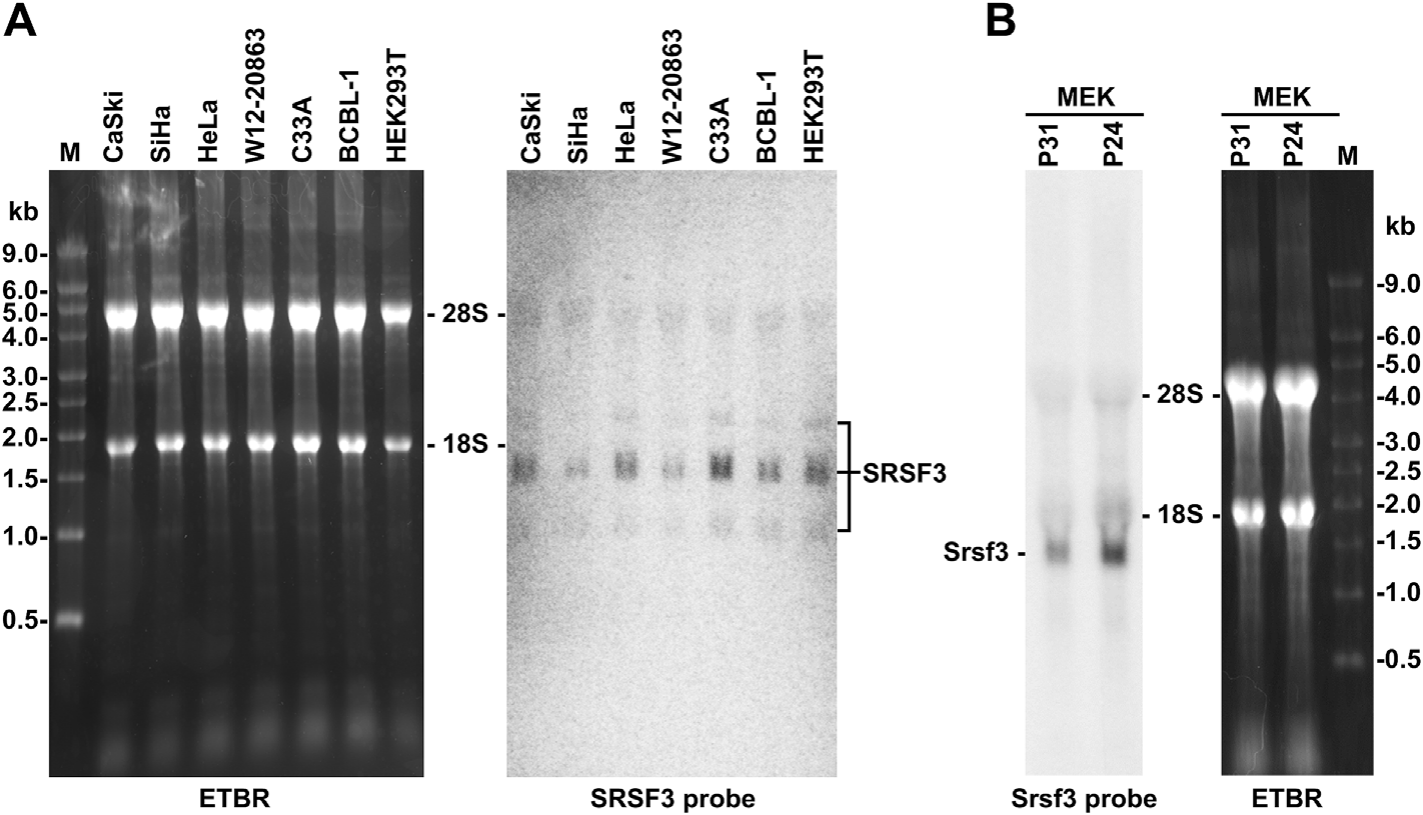
**Detection of human SRSF3 and mouse Srsf3 transcripts by Northern blot analysis**. Five micrograms of total RNA isolated from CaSki, SiHa, HeLa, W12–20863, C33A, BCBL-1, HEK293T cells and 10 μg of total RNA isolated from mouse epithelial keratinocytes (MEK) at passage 24 (P24) or passage 31 (P31) were separated by electrophoresis in 1% formaldehyde-denaturizing agarose gel together with the RNA Millennium Markers (Thermo Fisher Scientific). The ribosomal RNAs visualized by ethidium bromide staining (ETBR) were used as loading control. After transfer, the membrane was hybridized with ^32^P-labeled oligo probes antisense to exon 3 of human SRSF3 (A) or exons 1–3 of mouse Srsf3 (B). See Table 1 for probe sequence details.

An early report (Ayane et al., 1991) indicated that X16 (Srsf3 or SRp20) cDNA sequence from a mouse preB cell line 33.1.1 is 1383 bp long and contains a 492 bp open reading frame encoding 164 amino acid (aa) residues from the first AUG at nucleotide 63. The expressed major X16 mRNA in various tissues and cell types was measured in an estimated 1.6 kb by Northern blot analyses (Ayane et al., 1991). However, the major human SRSF3 transcript variant 1 from NCBI NM_003017.5 encodes the same ORF, but is surprisedly in size of 4228 nt and the mouse Srsf3 transcript NM_013663.5 from the mm10 reference genome is in size of 2585 nt. We noticed that the size difference between reported X16 cDNA sequence and the annotated NCBI SRSF3/Srsf3 sequences mainly occurs from the last exon, exon 7 of SRSF3/Srsf3, despite that a slight difference by a few nucleotides can be seen in the exon 1. Although the reported 3ʹ UTR region of X16 exon 7 was in size of 827 nt (Ayane et al., 1991), the annotated exon 7 3ʹ UTR in NCBI Srsf3 RefSeq NM_013663.5 encoding the full length Srsf3 protein is in size of 1961 nt and human SRSF3 RefSeq NM_003017.5 is 3611-nt long. To determine the accuracy of SRSF3 transcripts in mammalian cells, total RNA extracted from 7 human cell lines (CaSki, SiHa, HeLa, and C33A are cervical cancer cell lines; BCBL-1 is a body-cavity-based lymphoma cell line; W12-20863 is a subclone of the W12 cell line derived from a low-grade cervical lesion; HEK293T is an adenovirus-5 DNA-transformed normal human embryonic kidney cells) and normal mouse epithelial keratinocytes (MEK) at passage 24 (P24) and passage 31 (P31) was analyzed by Northern blot using ^32^P-labeled antisense oligo probes, respectively, targeting first three constitutive exons (exon 3 for human SRSF3 and exons 1–3 for mouse Srsf3). In human cells, the SRSF3 probes detected a major SRSF3 mRNA band slightly bigger than 1.5 kb and two other weak bands, one just above 1 kb and the other above 2 kb (Fig. 1A). Consistently with previous observation (Ayane et al., 1991), we found that the major isoform of the mouse Srsf3 RNA transcripts is less than ∼1.5 kb (Fig. 1B).

### 2.2 Mapping of the transcription start sites (TSS) for expression of human SRSF3 and mouse Srsf3

**Fig. 2:**
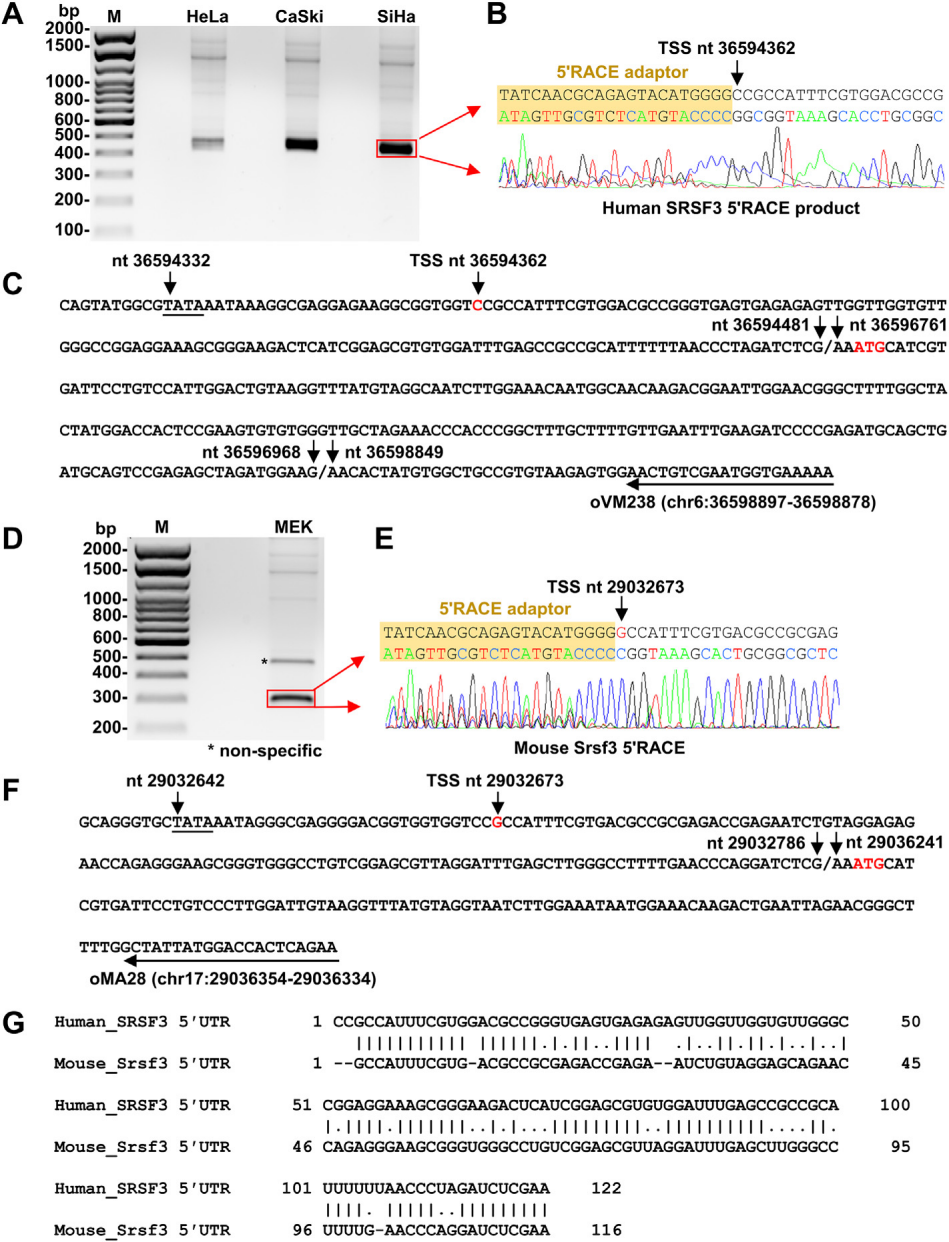
**Mapping of both human SRSF3 and mouse Srsf3 transcription start site (TSS)**. (A–C) Mapping of human SRSF3 TSS. A 5′ RACE was conducted with a human SRSF3-specific primer, oVM238 (located on SRSF3 exon 3), on total RNA isolated separately from three human cell lines, HeLa, CaSki, and SiHa (A). One major band from the 5ʹ RACE products in red rectangle (A) was gel purified and confirmed by Sanger sequencing (B). Human SRSF3 TSS was mapped to the hg38 chr6 nt 36594362 (in red) (B), which is 26 ​nt downstream of the TATA box (C). The entire sequence of the 5′ RACE region of human SRSF3 is shown along with a conserved TBP-binding site TATA box upstream of the mapped TSS position, the exon/exon junction positions, and the translation initiation codon ATG (red) (C). (D–F) Mapping of mouse Srsf3 TSS. A 5ʹ RACE was applied to mapping of mouse Srsf3 TSS on total RNA from mouse epithelial keratinocyte (MEK cells) (D) with a mouse Srsf3-specific primer oMA28 (located on exon 2) (F). Two major RACE products were gel purified (D) and sequenced by the Sanger sequencing (E). The products in the upper band (∗) turned to be a non-specific amplification. The lower band in red rectangle (D) is a specific 5ʹ RACE product with the mouse TSS (in red) being mapped at the mm10 chr17 nt 29032673 (E). The entire sequence of the 5ʹ RACE region of mouse Srsf3 is shown along with a conserved TBP-binding site TATA box 27 ​nt upstream of the mapped TSS position, exon 1/exon 2 junction positions, and the translation initiation codon ATG (red) (F). (G) The alignment showing the ∼70% homology between mapped SRSF3 and Srsf3 5ʹ UTRs.

Given the detected SRSF3 RNA size is much less than the NCBI NM_003017.5 and NM_013663.5, we mapped the transcription start sites (TSS) of human SRSF3 and mouse Srsf3. Total cell RNA extracted, separately, from three human cancer cell lines, HeLa, CaSki and SiHa (for human SRSF3) and mouse epithelial keratinocytes (MEK) (for mouse Srsf3) was used for 5′ RACE in the presence of a human SRSF3-specific antisense primer, oVM238 or a mouse Srsf3-specific antisense primer, oMA28. The 5′ RACE products were visualized by agarose gel electrophoresis (Fig. 2A and Fig. 2D).

As shown in Fig. 2B, one major band of the 5ʹ RACE products from all three human cell lines were gel-purified and sequenced. We found that human SRSF3 transcription is started at a pyrimidine C, the first C of a hexamer CCGCCA, at nt 36,594,362 on chr6 (Fig. 2B), not a purine A or G as reported for eukaryotes (Butler and Kadonaga, 2002). The human SRSF3 TSS we found is consistent with the annotated NCBI human SRSF3 gene (Gene ID: 6428, NM_003017.5).

Sequencing of the major band from the 5ʹ RACE products derived from MEK (Fig. 2D) showed that mouse Srsf3 transcription is initiated from a purine G, the third G of the same hexamer CCGCCA, at nt 29,032,673 on chr17 (Fig. 2E). This TSS is 13 nt downstream from NCBI annotated mouse Srsf3 gene (Gene ID: 20383, NM_013663.5), which starts at nt 29,032,660 on chr17. However, our result is consistent with the previous publication (Jumaa et al., 1997).

Analyses of the region upstream of human SRSF3 (Fig. 2C) and mouse Srsf3 TSS (Fig. 2F) show a TATA box (a eukaryotic core promoter motif) 26 bp upstream of the mapped human SRSF3 TSS and 27 bp upstream of the mapped mouse Srsf3 TSS, suggesting that the mapped TSS are authentic and highly conserved in the human and mouse genomes.

SRSF3 protein sequence is well conserved almost in all animal species. Translation of both human SRSF3 and mouse Srsf3 proteins is initiated from the first AUG in their RNA exon 2. This translation initiation codon bears the Kozak content with a purine G at −3 position (Fig. 2C and F) (Kozak, 1986, 1999). Although our mapped human SRSF3 TSS is consistent with the annotated SRSF3 TSS from the NCBI RefSeq NM_003017.5 sequence and has a 5′ UTR in size of 122 nt, the mapped mouse Srsf3 TSS is 13 nt shorter from the annotated Srsf3 TSS from the NCBI RefSeq NM_013663.5 and has a 5ʹ UTR in size of 116 nt. By comparing the 5′ UTR sequence between human SRSF3 and mouse srsf3 (Fig. 2G), we found they are ∼70% homology.

### 2.3 Mapping of the polyadenylation cleavage sites for expression of human SRSF3 and mouse Srsf3

**Fig. 3:**
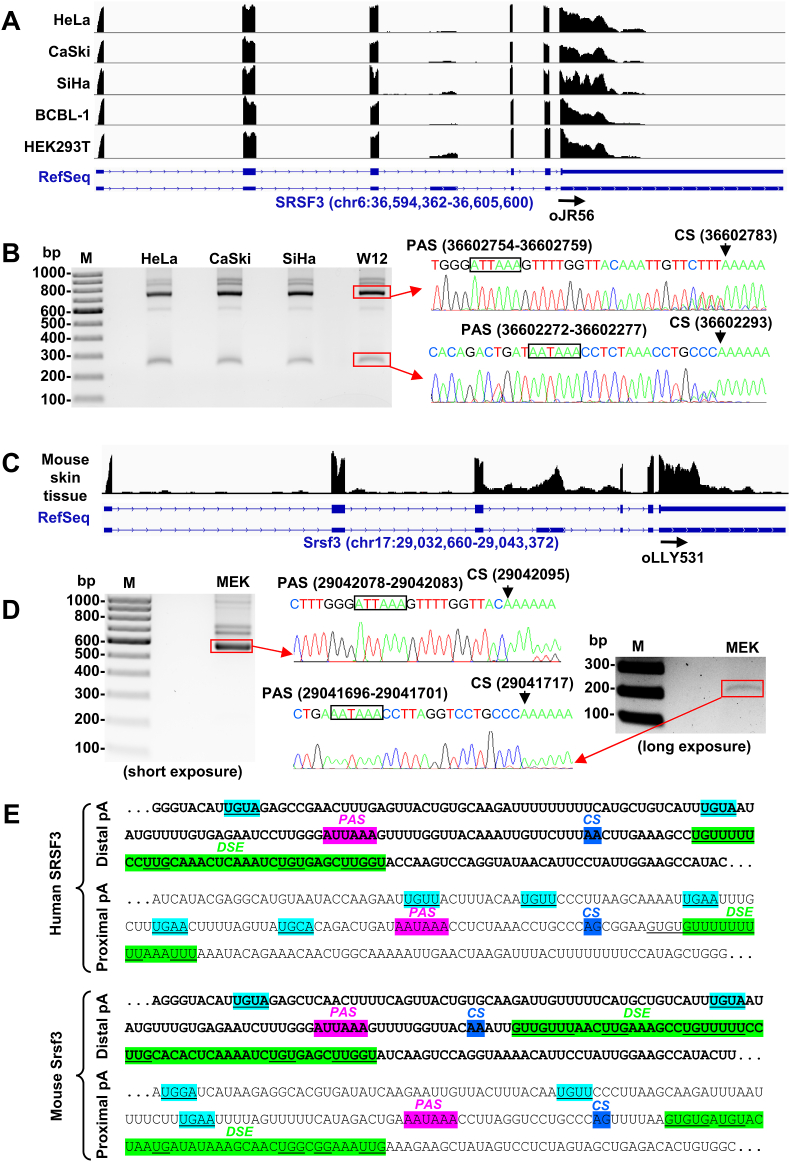
**Mapping of human SRSF3 and mouse Srsf3 RNA polyadenylation cleavage sites**. (A) Coverage and distribution of human SRSF3 RNA-seq reads from five different human cells along with human reference genome (hg38) by IGV visualization. An arrow to the right underneath the SRSF3 RefSeq is an oligo primer oJR56 from the terminal exon used for 3′ RACE in the panel B. (B) Mapping of human SRSF3 polyadenylation cleavage site by 3′ RACE and sequencing. A 3ʹ RACE was conducted with a human SRSF3-specific primer, oJR56, from the terminal exon (A), on total RNA isolated from three human cancer cell lines, HeLa, CaSki, SiHa and a human cervical keratinocyte cell line W12. Two major bands from 3ʹ RACE products in red rectangle were gel purified (left) and sequenced by the Sanger sequencing (right). The sequence reading on the right shows the boxed polyadenylation signal (PAS) and the mapped PA cleavage sites (CS) of human SRSF3 with the hg38 genome position labeled on the top. (C) Coverage and distribution of mouse Srsf3 RNA-seq reads from mouse skin tissues along with the mouse reference genome mm10. (D) Mapping of mouse Srsf3 polyadenylation cleavage site by 3′ RACE and sequencing. A 3ʹ RACE was performed using a mouse Srsf3-specific primer, oLLY531, from the terminal exon (arrow to the right in the panel C), on total RNA from mouse primary keratinocyte MEK cells. Similar to human cell 3ʹ RACE products, two major bands in red rectangle, a bigger band on the left panel with short exposure and a smaller band on the right panel with longer exposure, were gel purified and sequenced by the Sanger sequencing. The sequence reading in the middle panel shows the boxed PAS and mapped PA cleavage sites of mouse Srsf3 RNA with the labeled mm10 genome positions. (E) Identification of the core cis-elements upstream and downstream of the mapped SRSF3/Srsf3 polyadenylation signal and cleavage sites. Both human SRSF3 and mouse Srsf3 utilize AUUAAA as a major PAS for its RNA cleavage and polyadenylation, whereas a highly conserved AAUAAA hexamer pA signal further upstream only serves as a minor PAS for its RNA cleavage and polyadenylation. Both mapped cleavage sites are positioned 10–30 nt (highlighted in blue) downstream of PAS. The UGUA upstream of PAS (cyan) and G/U- or U-rich downstream sequence element (DSE) (green) located ∼10–30 nt downstream of the cleavage sites are also shown.

By searching RNA polyadenylation signals in the 3ʹ UTR regions within exon 7 of NCBI annotated SRSF3/Srsf3 RNAs (NM_003017.5 and NM_013663.5), we found several consensus polyadenylation signals (PAS) and wished to determine why these consensus PAS could not be used for RNA polyadenylation. We first mapped the RNA-seq reads from different human cell lines including HeLa, CaSki, SiHa, BCBL-1 and HEK293T to the annotated human SRSF3 gene in the human reference genome hg38 and visualized distribution of the SRSF3 RNA-reads along with the annotated SRSF3 gene by the Integrative Genomics Viewer (IGV, The Broad Institute). We discovered that the RNA-reads from all cell lines were consistently mapped only to the first one-third of the exon 7. The last two-third of exon 7 annotated by the NCBI NM_003017.5 exhibited no mapped RNA-reads (Fig. 3A), indicating no RNA expression from this genome region. Similar SRSF3 reads-distributions were observed in RNA-seq from 54 normal human tissues analyzed by Genotype-Tissue Expression (GTEx) Program and 20 of them are selectively shown in Fig. S1.

To determine the real SRSF3 cleavage sites (CS) for SRSF3 RNA polyadenylation in the human genome, total RNA isolated from HeLa, CaSki, SiHa, and W12 cells was analyzed by 3′ RACE using a SRSF3-specific primer oJR56 from the beginning of exon 7 (Fig. 3A). Following gel purification and sequencing of the major 3′ RACE products (Fig. 3B), we found the strong upper band (Fig. 3B) was a product with a 3′ end at nt 36,602,783 on chr6, 23 nt downstream of a PAS AUUAAA at nt 36,602,754, and the weak lower band (Fig. 3B) was a product with a 3′ end at nt 36,602,293 on chr6, 15 nt downstream of a classical PAS AAUAAA at nt 36,602,272.

Similar to human SRSF3, RNA-seq reads from mouse skin tissues were mapped to the annotated Srsf3 gene (Gene ID, 20383; NM_013663.5) in the mouse reference genome mm10 and visualized their distribution along with the annotated Srsf3 gene by IGV. We discovered again the similar discrepancy of RNA-seq reads distribution in the Srsf3 exon 7 to that of human SRSF3, with Srsf3 RNA-seq reads only being mapped to the two-third of Srsf3 exon 7 (Fig. 3C).

To determine the authentic 3ʹ end of mouse Srsf3 RNA transcripts, total RNA from MEK cells was subjected to 3ʹ RACE using a Srsf3-specific primer, oLLY531 (Fig. 3C). Following gel purification and sequencing of the two major bands from 3′ RACE products, we found that the strong upper band was a product with a 3′ end at nt 29,042,095 on chr17, 11 nt downstream of a PAS AUUAAA at nt 29,042,078, indicating that most of the mouse Srsf3 transcripts are cleaved at the nt 29,042,095 for polyadenylation (Fig. 3D). Moreover, sequencing of the weaker lower band showed that fewer of mouse Srsf3 transcripts could be cleaved at the nt 29,041,717 for RNA polyadenylation by using a PAS AAUAAA at nt 29,041,696, 15 bp upstream of the cleavage site (Fig. 3D).

RNA polyadenylation is essential for gene expression, which is initiated by cleavage of the nascent transcripts and followed by addition of ∼150 adenosine residues at the RNA 3′ end. RNA transcripts without a poly(A) tail are degraded or not efficiently exported to the cytoplasm. Efficient RNA polyadenylation requires four core cis elements: an UGAU cis-element for a downstream PAS definition, a cleavage site generally positioned 10–30 nt downstream of PAS, and a U/GU-rich motif downstream (Gilmartin, 2005; Jurado et al., 2014). As shown in Fig. 3E, all the identified 3ʹ ends of human SRSF3 and mouse Srsf3 transcripts are surrounded by these four core elements and thus the polyadenylation cleavage sites we mapped are authentic.

### 2.4 The 3ʹ untranslated regions of human SRSF3 and mouse Srsf3 and their miRNA seed match

**Fig. 4:**
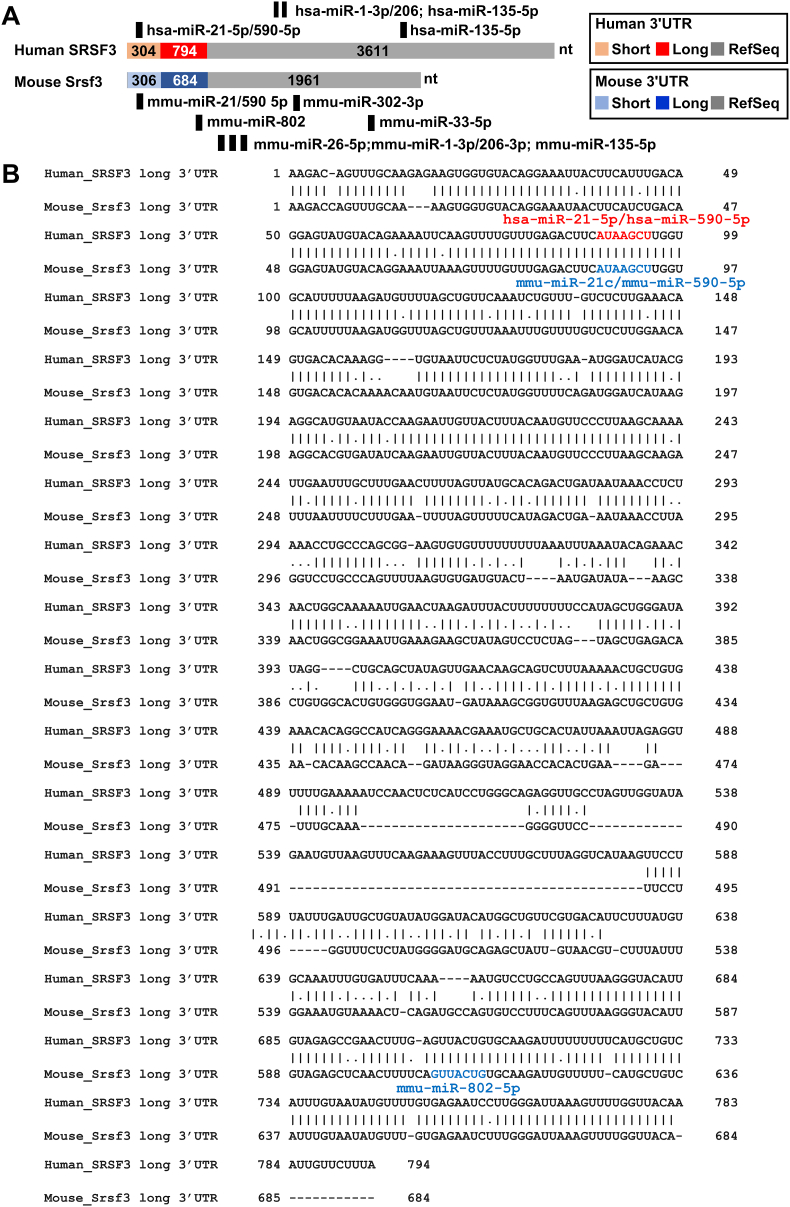
**Potential regulation of SRSF3/Srsf3 expression by 3ʹ UTR-targeting miRNAs.** (A) The conserved miRNA binding sites (seed matches) based on TargetScan in the RefSeq annotated (grey) human SRSF3 3ʹ UTR in size of 3611 nt and mouse Srsf3 3ʹ UTR in size of 1961 nt or the mapped 3ʹ UTRs of human SRSF3 (red-794 nt long UTR, pink-304 nt short UTR) and mouse Srsf3 (blue −684 nt long UTR, light blue −306 nt short UTR). Vertical black bars above the human SRSF3 3ʹ UTR or below the mouse Srsf3 3ʹ UTR indicate the miRNA-binding sites predicted by TargetScan program (https://www.targetscan.org/vert_80/). (B) Alignment of the mapped long 3ʹ UTR of human SRSF3 and mouse Srsf3 in this study and their potential miRNA binding sites predicted by TargetScan. Human hsa-miRNA binding site are labeled in red color and mouse mmu-miRNA binding sites are in blue color.

As described above, the mapped 3ʹ UTRs from human SRSF3 and mouse Srsf3 RNAs are much shorter than the annotated 3ʹ UTRs from the NCBI RefSeq SRSF3/Srsf3 sequences. The major isoform of human SRSF3 RNA we identified has a 3ʹ UTR only in size of 794 nt, not 3611 nt as annotated from the NCBI NM_003017.5 (Fig. 4A). The major isoform of mouse Srsf3 RNA 3ʹ UTR we determined is 684-nt long, not in size of 1961 nt annotated from the NCBI NM_013663.5 (Fig. 4A). By comparing the 3ʹ UTR sequences between human SRSF3 (794 nt) and mouse srsf3 (684 nt), we found that they share ∼70% homology (Fig. 4B).

The 3ʹ UTR has been a major focus in functional miRNA studies (Bartel, 2009) and the number of miRNA seed matches (binding sites) (Lewis et al., 2005) identified in the 3ʹ UTR of a given mRNA is positively correlated to miRNA-mediated translational repression (Mayr and Bartel, 2009) although functional miRNA binding sites are also found in the coding region (Kang et al., 2011; Schnall-Levin et al., 2011; Agarwal, Bell, Nam, & Bartel, 2015). Using miRNA TargetScan search program, we identified both human SRSF3 and mouse Srsf3 3ʹ UTRs contain a conserved miRNA binding site (Mayr, 2017) AUAAGCU for binding of miR-21-5p and miR-590-5p (Fig. 4A and B). In addition, the mouse Srsf3 3ʹ UTR has another miRNA seed match for binding of mmu-miR-802-5p which is missing from human SRSF3 3ʹ UTR (Fig. 4A and B). The data suggest that human SRSF3 and mouse Srsf3 might be regulated differentially in different tissues by available miRNAs in a different context.

### 2.5 Revised RNA isoforms of human SRSF3 and mouse Srsf3

**Fig. 5:**
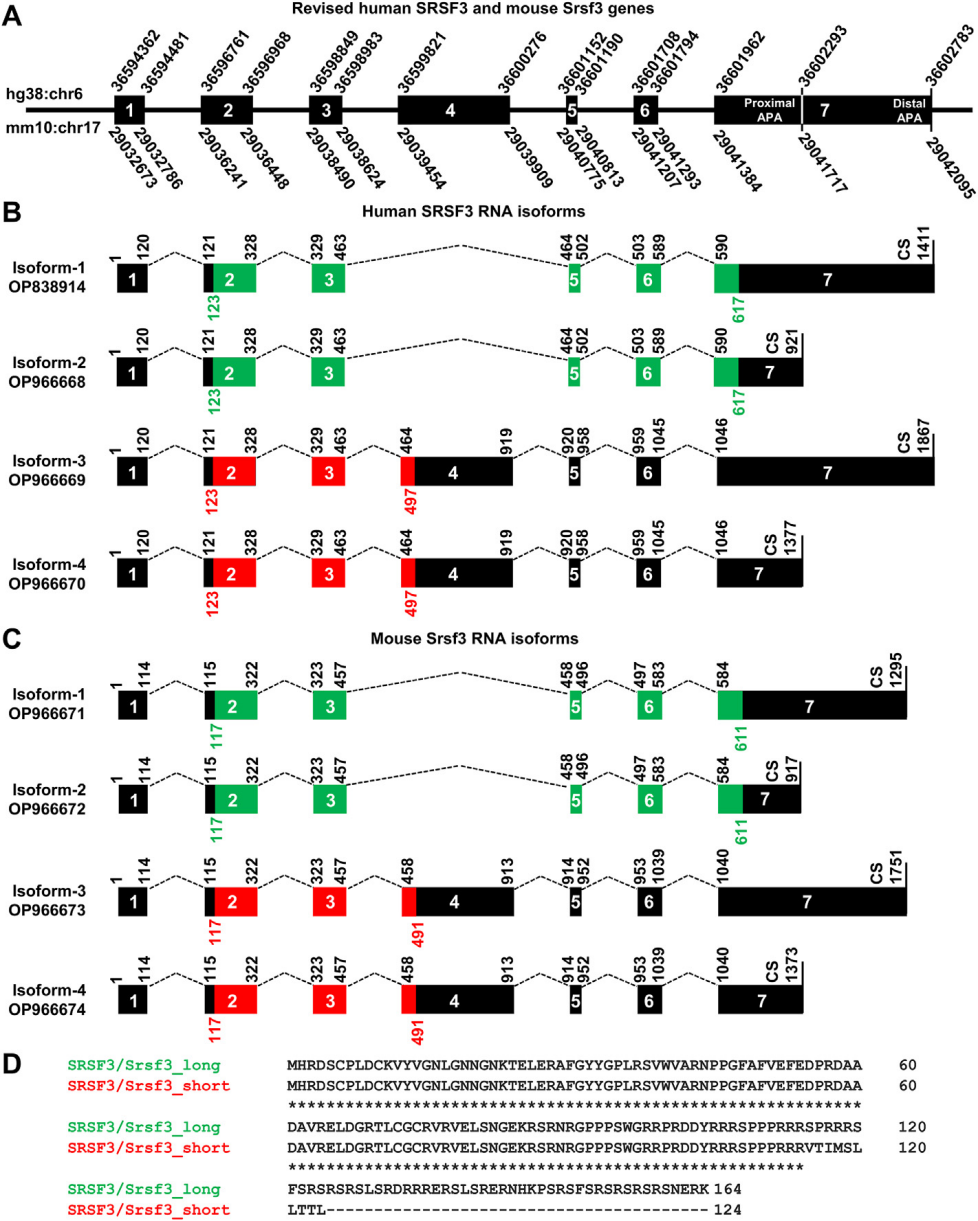
**Revised mRNA structures of human SRSF3 and mouse Srsf3**. (A) Revised gene structure of human SRSF3 and mouse Srsf3 from this report including genomic positions in the reference human (hg38) and mouse (mm10) genomes. (B and C) Revised RNA isoforms and their individual NCBI accession numbers (OP numbers) of human SRSF3 and mouse Srsf3. Both human SRSF3 and mouse Srsf3 contains 7 exons and 6 introns. Exon 4 is an alternative exon and contains a pre-mature termination (PMT) codon. Inclusion of exon 4 in spliced SRSF3 triggers nonsense-mediated decay of the exon 4-containing SRSF3 mRNA. Due to alternative exon 4 splicing and alternative PAS usage, four RNA isoforms of human or mouse SRSF3 will be produced, respectively, either from the human or mouse genome. The full-length SRSF3 mRNA without exon 4 is predominant and encodes SRSF3 protein of 164 amino acid residues (ORF in green), whereas the exon 4-bearing RNA, if not degraded, encodes a truncated protein of 124 aa residues (ORF in red). (D) The alignment of long (164 aa) and short (124 aa) SRSF3 or Srsf3 protein.

Based on the mapped TSS and polyadenylation cleavage sites of human SRSF3 and mouse Srsf3, we are able to revise the gene structure of both human SRSF3 and mouse Srsf3 (Fig. 5A) and identify four RNA isoforms from each animal species (Fig. 5B and C). Human SRSF3 primary transcripts span over 8422 bases as we demonstrated in this study, not 11,248 bases as annotated in human genome GRCh38/hg38 (chr6:36,594,353–36,605,600) (https://www.genecards.org/cgi-bin/carddisp.pl?gene=SRSF3). We mapped SRSF3 TSS at nt 36,594,362 on chr6 and its primary RNA transcripts are cleaved at nt 36,602,783 mainly using a distal PAS at nt 36,602,754 to produce the SRSF3 pre-mRNAs in size of 8422 nt, but uncommonly cleaved at nt 36,602,293 by using a proximal PAS at nt 36,602,272. This alternative polyadenylation of human SRSF3 pre-mRNAs produces a minor isoform in size of 7932 nt, 490 nt shorter than the major longer isoform (Fig. 5A and B).

Mouse Srsf3 transcription is started at nt 29,032,673 on chr17 and its primary RNA transcripts are polyadenylated also at two alternative pA sites, with the major isoform cleaved at nt 29,042,095 using a distal PAS at nt 29,042,078 and the minor isoform cleaved at nt 29,041,717 using a proximal PAS at nt 29,041,696. Thus, the major Srsf3 pre-mRNAs are 9423-nt long and the minor Srsf3 pre-mRNA has 9045 nt in size which is 378 nt less than the longer isoform (Fig. 5A and C).

There are 7 exons and 6 introns in both human SRSF3 and mouse Srsf3 pre-mRNAs. However, the 456-nt long exon 4 of both human SRSF3 and mouse Srsf3 is an alternative “poison” exon containing a pre-mature termination codon (PMT) and is normally excluded from a mature mRNA during pre-mRNA splicing. Given alternative RNA polyadenylation, therefore, four mRNA isoforms are produced from human SRSF3 or mouse Srsf3 pre-mRNA processing and illustrated, respectively, in Fig. 5B and C based on this study. Human SRSF3 mRNA isoform-1 with 1411 nt in Fig. 5B and mouse Srsf3 mRNA isoform-1 with 1295 nt in Fig. 5C are the two major RNA isoforms and easily detectable by Northern blot (Fig. 1A and B), which code full-length SRSF3 protein with 164 aa residues (Fig. 5D). The minor RNA isoforms of SRSF3 and Srsf3 from proximal PAS usage are only in size of 921 nt and 917 nt, respectively and also translate the full length SRSF3 protein. A Northern blot weak band just above 1 kb detected from human cells could be the isoform-2 of the minor SRSF3 mRNA (Fig. 1A). The RNA isoforms with exon 4 (456 nt) retention subject to nonsense-mediated RNA decay (NMD) (Guo et al., 2015) and encode a truncated protein of 124 aa residues if detectable (Fig. 5D). In fact, a Northern blot weak band just above ∼2 kb detected from human cells could be the remaining isoform-3 of SRSF3 mRNA (Fig. 1A).

## 3 Discussion

SRSF3 is one of most important splicing factors involving in many cellular functions (Jia et al., 2009, 2019; Majerciak et al., 2014; Ajiro et al., 2016) and SRSF3 overexpression is oncogenic (Jia et al., 2010; He et al., 2004, 2011). Although the size of mouse Srsf3 RNA was originally identified in ∼1.5 kb by Northern blot (Ayane et al., 1991), we found that human SRSF3 RefSeq (NCBI NM_003017.5) and mouse Srsf3 RefSeq (NCBI NM_013663.5) have a much larger size of the sequence, 4228 nt for human SRSF3 and 2585 nt for mouse Srsf3. In this report, we confirmed the early report by Northern blot of mouse Srsf3 in ∼1.5 kb. We further found by Northern blot that human cells express three detectable SRSF3 RNA isoforms and the major isoform-1 (∼67%) of SRSF3 RNA is ∼1.5 kb. The other two minor isoforms of SRSF3 are ∼1.0 kb of the isoform-2 (∼20%) and ∼2 kb of the isoform-3 (∼13%) (Fig. 1A), with a variable fraction of the isoform-3 production from different cell types. By mapping of the TSS and RNA polyadenylation cleavage site of SRSF3, we redefined the major human SRSF3 RNA isoform in size of 1411 nt, and the major mouse Srsf3 RNA isoform in size of 1295 nt, of which addition of ∼150 poly A nucleotides makes each in size close to ∼1.5 kb in Northern blot. The difference from the redefined RNA size of SRSF3 or Srsf3 to the corresponding RefSeq sequence is limited to the terminal exon 7 and mainly constrained at the 3ʹ UTR region.

The RNA 3ʹ UTR has been extensively explored for its regulatory functions of RNA polyadenylation, RNA stability, RNA-protein and -miRNA interactions, subcellular mRNA location and protein translation (Mignone et al., 2002; Moore, 2005; Mayr, 2019). It has been observed that cancer cell lines often express substantial amounts of mRNA isoforms with shorter 3ʹ UTRs to escape miRNA-mediated repression of protein translation (Mayr and Bartel, 2009). Our findings indicate that human SRSF3 and mouse Srsf3 have a much shorter 3ʹ UTR than the annotated RefSeq sequence in the NCBI database. Our data raise a serious concern to those who heavily depend on bioinformatics analyses on the NCBI RefSeq sequences to draw a “big conclusion” or “discovery”. The similar wrong annotations in the KSHV genome based on computer predictions (Russo et al., 1996; Neipel et al., 1997) and functional studies (Burýsek and Pitha, 2001) had been happened initially to vIRF-2, ORF50 and ORF57 where the authentic viral ORFs were later identified being spanning over two different exons at different genome locations (Cunningham et al., 2003; Lukac et al., 1999; Kirshner et al., 2000; Majerciak et al., 2006; Sharma et al., 2017). To avoid the future misleading publications purely based on these wrongful annotations, we strongly suggest that a careful validation of the annotated RefSeq sequence of the interested gene should be necessary ahead of moving the research project forward.

A widespread mechanism to generate mRNA isoforms with alternative length of the 3ʹ UTRs is by RNA alternative cleavage and polyadenylation (APA) which has emerged as a major regulator of mRNA fate and functions of protein synthesis (Moore, 2005; Mitschka and Mayr, 2022). We discovered in this report that the expression of both human SRSF3 and mouse Srsf3 genes is undertaken by APA either using a preferential distal AUUAAA PAS to produce SRSF3 or Srsf3 with a longer 3ʹ UTR or an unfavorable proximal AAUAAA PAS to generate SRSF3 or Srsf3 with a shorter 3ʹ UTR (Fig. 4, Fig. 5). Although the proximal AAUAAA PAS is a highly conserved PAS over the variant distal AUUAAA in mammalian RNA polyadenylation, APA selection of the distal variant PAS to generate the longer 3ʹ UTR in the expression of human SRSF3 or mouse Srsf3 has to run through the proximal PAS. Currently, the mechanism in preferential usage of the distal AUUAAA PAS over the proximal AAUAAA PAS remains unknown.

## 4 Materials and methods

### 4.1 Cell lines

CaSki, SiHa, HeLa, C33A and HEK293T cells were obtained from ATCC (Manassas, VA) and were maintained in Dulbecco's modified Eagle's medium (DMEM) (Thermo Fisher Scientific, Waltham, MA) with 10% fetal bovine serum (FBS, GE Healthcare, Logan, UT). BCBL-1 cells (Renne et al., 1996) were grown in the suspension in RPM1640 media (Thermo Fisher Scientific, Waltham, MA) supplemented with 10% fetal bovine serum (FBS). W12 -20863 cells were cultured in F-medium (3:1 (v/v) F-12-DMEM, 5% FBS, 0.4 μg/ml hydrocortisone, 5 μg/ml insulin, 8.4 ng/ml cholera toxin, 10 ng/ml epidermal growth factor (EGF), 24 μg/ml adenine, 100 U/ml penicillin, 100 μg/ml streptomycin) in the presence of irradiated J2 3T3 feeder cells. Primary mouse keratinocytes (MEK) from newborn C57Bl/6NCr mouse were cultivated as described (Chapman et al., 2010; Yu et al., 2021) in the presence of Rho kinase inhibitor Y-27632 (Enzo Life Sciences, #ALX-270-333). All cells were cultivated at 37 °C under a 5% CO_2_ atmosphere.

### 4.2 Total RNA sequencing and bioinformatics

The total RNA extracted by TriPure reagent (Roche, #11667165001) was used for construction of RNA-seq libraries followed by Illumina sequencing. Sequencing libraries were constructed following Illumina Stranded Total RNA protocol (Illumina, RS-122-2201). Paired-end 150-bp read length sequencing with a depth of 100 million reads per sample was performed on the HiSeq 2500 sequencer according to the manufacturer's instructions (Illumina) for CaSki cells. For SiHa cells, the length of the paired-end read is 75-bp with a depth of 75 million reads per sample. For HeLa cells, the length of the paired-end read is 50-bp with a depth of 25 million reads per sample. For BCBL-1 cells, the length of the paired-end read is 150-bp with a depth of 40 million reads per sample. For mouse skin tissues, the length of the paired-end read is 126-bp with a depth of 50 million reads per sample. Raw data and analyzed RNA-seq data supporting the findings in this study have been deposited in the NCBI GEO database under each accession number: GSE158033, GSE179727, GSE179728, and GSE136647. The read were aligned to the reference genomes hg38 (human) and mm10 (mouse) using STAR aligner and the reads mapped to SRSF3/Srsf3 were visualized by the Integrative Genomics Viewer (IGV, https://software.broadinstitute.org/software/igv/). The 5ʹ and 3ʹ UTR were aligned using EMBOSS Stretcher (https://www.ebi.ac.uk/Tools/psa/emboss_stretcher/). The protein sequences were aligned using Clustal Omega (https://www.ebi.ac.uk/Tools/msa/clustalo/).

### 4.3 5ʹ and 3ʹ rapid amplification of cDNA ends (RACE)

Total RNA was extracted using the TriPure reagent (Roche, #11667165001). The 5′ and 3ʹ RACE assays were conducted with a SMARTer RACE cDNA amplification kit (Takara, #634858) as recommended with 1 μg/reaction of total RNA as a template. The following primers were used: human SRSF3 primer oJR56 was used for 3ʹ RACE PCR amplification, while primer oVM238 was used for 5ʹ RACE PCR amplification; mouse Srsf3 primer oLLY531 was used for 3ʹ RACE PCR amplification, and primer oMA28 was used for 5ʹ RACE PCR amplification. See Table 1 for each oligo primer sequence. The obtained RACE products were analyzed by agarose electrophoresis, gel purified and subjected to Sanger sequencing.

**Table 1:** **Oligos used in this study**.The oligo location is referring to the exon number in the RefSeq NM_003017.5 for human SRSF3 and NM_013663.5 for mouse Srsf3. RACE, rapid amplification of cDNA ends; NB, Northern blot.

Name	Sequence (5′-3′)	Genome	Chromosome position	Strand	Location	Assay
**oVM238**	TTTTTCACCATTCGACAGTT	hg38	chr6:36598897-36598878	–	exon 3	5′ RACE/NB
**oJR56**	TCTCTTGAAACAGTGACACAAAGGTG	hg38	chr6:36602126-36602151	+	exon 7	3′ RACE
**oMA28**	TTCTGAGTGGTCCATAATAGC	mm10	chr17:29036354–29036334	–	exon 2	5′ RACE/NB
**oLLY531**	GGCACGTGATATCAAGAATTGTTACT	mm10	chr17:29041610–29041635	+	exon 7	3′ RACE
**oMA229**	CGAGATCCTGGGTTCAAAAG	mm10	chr17:29032786–29032767	–	exon 1	NB
**oLLY537**	TTCCACTCTTACACGGCAGC	mm10	chr17:29038520–29038501	–	exon 3	NB

### 4.4 Northern blot

5–10 μg of total RNA was isolated from cells using TriPure Reagent (Roche) and separated in 1% formaldehyde-denaturing agarose gel together with the RNA Millennium Marker (ThermoFisher Scientific). The ribosomal RNAs visualized by ethidium bromide staining (ETBR) were used as loading control. After transfer, the membrane was hybridized with ^32^P-labeled oligo probes antisense to exon 3 of human SRSF3 or exons 1–3 of mouse Srsf3 (see Table 1 for details).

### 4.5 miRNA binding site prediction

The miRNA-binding sites in the 3ʹUTR of human SRSF3 and mouse Srsf3 mRNAs were predicted by Targetscan Release 8.0 (https://www.targetscan.org/vert_80/) using default setting (Agarwal, Bell, Nam, & Bartel, 2015; Mcgeary et al., 2019).

## Author contributions

L.Y., V.M., and Z.M.Z. designed the study. L.Y. and V.M. performed the experiments, bioinformatic analysis, and composed figures. L.Y., V.M., R.J. and Z.M.Z. contributed to data analyses and manuscript writing, editing, and proofreading. All authors have read and agreed to the final version of the submitted manuscript.

## Declaration of competing interest

The authors declare no conflict of interest.

## Appendix A Supplementary data

Multimedia component 1 The distribution of RNA-seq reads along with the RefSeq annotated SRSF3 gene in 20 selected human normal tissues from Genotype-Tissue Expression (GTEx) Program (https://commonfund.nih.gov/GTex) visualized in the same reads-scale (0–1626) by UCSC Genome Browser hg38 (https://genome.ucsc.edu/). Multimedia component 1
