# Supplementary material for: Revisiting and corrections to the annotated SRSF3 (SRp20) gene structure and RefSeq sequences from the human and mouse genomes
Source: Cell Insight. 2023 Feb 26;2(2):100089. doi: 10.1016/j.cellin.2023.100089 (PMC10134197; doi:10.1016/j.cellin.2023.100089)
Supplement: Multimedia component 1 — The distribution of RNA-seq reads along with the RefSeq annotated SRSF3 gene in 20 selected human normal tissues from Genotype-Tissue Expression (GTEx) Program (https://commonfund.nih.gov/GTex) visualized in the same reads-scale (0–1626) by UCSC Genome Browser hg38 (https://genome.ucsc.edu/). [file mmc1.pdf]

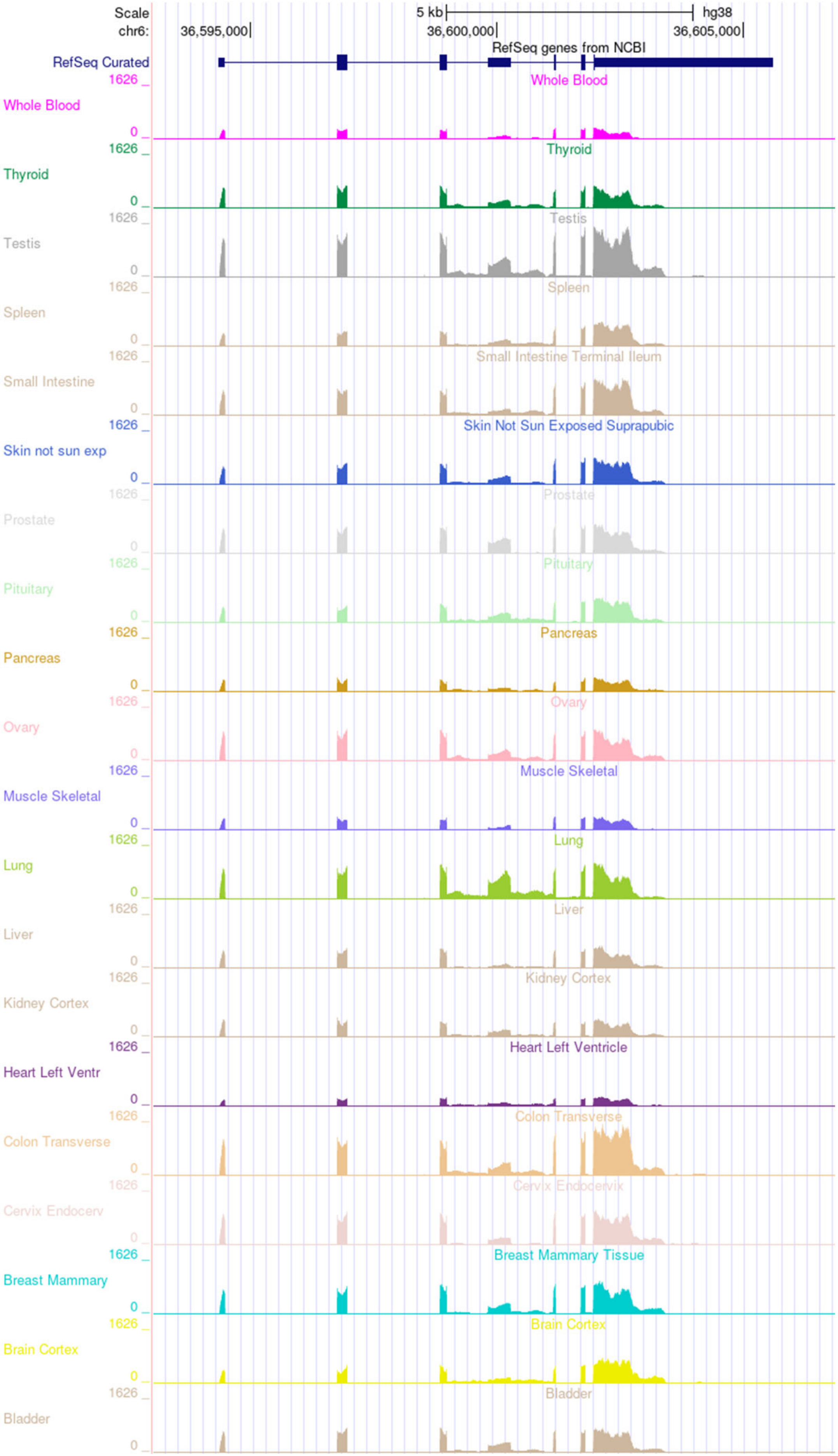

Fig. S1. The distribution of RNA-seq reads along RefSeq annotated SRSF3 gene in 20 selected human normal tissues from Genotype-Tissue Expression (GTEx) Program (https://commonfund.nih.gov/GTex) visualized in the same reads-scale (0-1626) by UCSC Genome Browser hg38 (https://genome.ucsc.edu/).
